# Does managed competition constrain hospitals' contract prices? Evidence from the Netherlands

**DOI:** 10.1017/S1744133119000215

**Published:** 2020-07

**Authors:** Rudy Douven, Monique Burger, Frederik Schut

**Affiliations:** 1 CPB, Netherlands Bureau for Economic Policy Analysis, The Hague, The Netherlands; 2 Erasmus School of Health Policy and Management, Erasmus University, Rotterdam, The Netherlands

**Keywords:** Contract prices hospitals, hospital–insurer negotiations, managed competition, price variation, I00, I11, L11, L51

## Abstract

In the Dutch health care system, health insurers negotiate with hospitals about the pricing of hospital products in a managed competition framework. In this paper, we study these contract prices that became for the first time publicly available in 2016. The data show substantive price variation between hospitals for the same products, and within a hospital for the same product across insurers. About 27% of the contract prices for a hospital product are at least 20% higher or lower than the average contract price in the market. For about half of the products, the highest and the lowest contract prices across hospitals differ by a factor of three or more. Moreover, hospital product prices do not follow a consistent ranking across hospitals, suggesting substantial cross-subsidization between hospital products. Potential explanations for the large and seemingly random price variation are: (i) different cost pricing methods used by hospitals, (ii) uncertainty due to frequent changes in the hospital payment system, (iii) price adjustments related to negotiated lumpsum payments and (iv) differences in hospital and insurer market power. Several policy options are discussed to reduce variation and increase transparency of hospital prices.

## 1. Introduction

Worldwide, policy makers are searching for new ways to organize hospital care efficiently. In most hospital markets, a nationwide product classification system exists that allows third-party payers to identify the product a patient receives and to compare productivity across hospitals. However, countries make different choices in how to price these hospital products (Siciliani *et al*., 2017).

One extreme option of pricing hospital products is fixed prices. For example, Germany, the UK and traditional Medicare in the US have a system with administered prices where prices are determined by the regulating authority. Competition among hospitals will take place in non-price means, like volume and quality (Gaynor *et al*., 2015).

The other extreme is that prices of individual hospital products are fully market-determined. In this situation, prices are determined through a bargaining process between health insurers (or other third-party payers) and hospitals. Some examples approaching this extreme are the employer-based insurance market and Medicare Advantage in the US, and systems of managed competition in Switzerland and the Netherlands. Competition among hospitals and insurers will then be based on prices, volume and quality (Gaynor *et al*., 2015).

It is still an open question whether a situation of fixed hospital prices is preferable over a situation with largely market-determined prices, or *vice versa*. Outcomes of different hospital pricing systems are difficult to compare or evaluate and depend on many different factors that are often hard to observe and difficult to isolate. In a market where prices are negotiated by health insurers and hospitals, important preconditions are that health insurers can act as a prudent buyer of care for their consumers and can steer their patients to hospitals with a good price–quality ratio (Enthoven, 1993).

In the Netherlands, there is a system of managed competition where insurers negotiate with hospitals about individual prices for a large share of the hospital products. For a long time, the contract prices of these hospital products were considered as private information and were not publicly available, making it difficult to evaluate pricing of hospital products. However, in 2016, in response to growing pressure from consumer associations, one large insurer and one hospital group made their contract prices publicly available. This allowed us to study negotiated prices of hospital products for the first time.

We hypothesize that in a good functioning managed competition framework, hospital–insurer negotiations will lead to low price variation for similar products. The reason is that health insurers –all else equal– will use comparative information on hospital prices to negotiate the lowest possible prices and will motivate their enrollees to visit the lowest priced hospitals, which will drive down price variation across similar hospital products. Furthermore, we expect that hospitals would charge similar prices for the same products to different health insurers if there is effective competition among insurers. The aim of this paper is to examine the extent of price variation between hospitals for the same hospital products, and within a hospital for the same products across insurers by analyzing publicly available price information.

We find a substantive price variation. About 27% of the contract prices for a hospital product are at least 20% higher or lower than the average contract price in the market. For about half of the products, the highest and the lowest contract prices across hospitals differ by a factor of three or more. Such large price variations for similar products cannot be explained by underlying differences in price–cost margins, and therefore indicate the presence of substantial cross-subsidization between hospital products within hospitals. In addition, we find evidence of considerable price discrimination across insurers as the prices of the same product charged by a single hospital on average vary by a factor of 1.4. Since these differences are not consistent across different hospital products, this price variation cannot solely be explained by differences in (regional) buying power of insurers. Moreover, since the prices charged to different insurers vary for most of the products, the way prices are set seems to differ per insurer. Below we discuss several potential explanations for these findings.

Empirical studies based on actual contract prices that are negotiated between hospitals and insurers in a market with managed competition are scarce. An important reason is that data on contract prices are often proprietary and confidential. Therefore, researchers have to rely on hospital list prices (i.e. non-contracted prices that are set by hospitals themselves and have to be paid by uninsured patients and by patients visiting non-contracted hospitals) or other proxies of contract prices, which can substantially differ from the true contract prices (Halbersma *et al*., 2011). Based on insurance claims data for individuals with private employer-sponsored health insurance in the US, Cooper *et al*. (2015) found a large variation in overall inpatient hospital prices and in prices for seven relatively homogenous procedures. In the Netherlands, there are some earlier studies using proprietary data. Halbersma *et al*. (2011) studied the initial year, 2005, of free hospital–insurer price negotiations in the Netherlands. They found a significant impact of idiosyncratic effects on hospital prices, which, as argued by the authors, is consistent with the fact that the Dutch hospital sector is not yet in a long-run equilibrium. Heijink *et al*. (2013) studied a single hospital procedure (i.e. cataract surgery) and found evidence of substantial price variation that was persistent over time (from 2006 to 2009). Our study is the first for the Netherlands covering a comprehensive set of hospital contract prices using the recently published data on hospital prices.

The paper is organized as follows. In Section 2, we discuss the main features of the Dutch hospital market and the price setting of hospital products. Data and methods are described in the third section. The results are described in the fourth section and potential explanations are discussed in the fifth section. The final section discusses the policy implications of our findings that may not only be relevant for the Netherlands but also for other countries that have implemented, or are intending to implement, market-determined hospital prices.

## 2. Price setting in the Dutch hospital market

The Dutch hospital market is gradually changing from a supply-side-regulated to a more demand-side-driven system. In 2006, the Health Insurance Act (HIA) came into force introducing market mechanisms as instruments for cost control. For hospitals, contractual negotiations with insurers about price and quality of their services are central in this reform. The intention of the government was to gradually step back in regulating the capacity and prices of health care supply and leave it more to the market (Schut and Van de Ven, 2011).

However, the introduction of such a system takes time, and since 2005, the hospital payment system has been frequently and profoundly changed (see Table 1).

**Table 1.** Overview of important changes in the hospital payment system since 2005

Year	Change
2005	Introduction of DTC product classification system (>40,000 DTCs)
	Introduction of prices per DTC instead of hospital per diem rates
	Introduction of freely negotiable prices for on average 10% of hospital revenue
2008	Expansion of free pricing segment to on average 20% of hospital revenue
2008–2017	Phasing out retrospective capital cost compensations
2009	Expansion of free pricing segment to on average 34% of hospital revenue
2012	Profound revision (DOT) of DTC product classification system (4400 DTCs)
	Expansion of free pricing segment to on average 70% of hospital revenue
2012–2014	Phasing out global hospital budgeting system (transition model)
2012–2018	General agreements about macro budget total hospital expenses
	Insurers and hospitals negotiate about lumpsum payments and DTC prices
2015	Integration of medical specialist remuneration in DTC prices
	Reduction of maximum duration of DTCs from 365 to 120 days

To facilitate contractual negotiations at a product level, in 2005 all hospital services were classified into a diagnosis treatment combination (DTC) (Oostenbrink and Rutten, 2006; Westerdijk *et al*., 2011). Each DTC included all hospital activities and services (both inpatient and outpatient) associated with the patient's demand for care, from the initial consultation to the final check-up. At the same time, the system of hospital pricing based on per diem rates was replaced by a system of prices per DTC.

Initially, most of these DTC prices were regulated and only for a minority of routine elective hospital treatments (e.g. cataract surgery and hip replacements), DTC prices were made freely negotiable between hospitals and insurers. In due course, the average share of freely negotiable DTCs was stepwise expanded, from about 10% of hospital revenue in 2005 to 70% in 2012. For the remaining 30% (consisting of the most complex hospital products), prices are still regulated. The introduction of DTCs was accompanied with a gradual phasing out of retrospective capital cost compensations for hospitals, making hospitals increasingly at risk for investments in hospital infrastructure.

In 2012, the DTC system was simplified to reduce the complexity, administrative costs and incentives for upcoding, by reducing the number of DTCs, from over 40,000 products to about 4400 products (Krabbe-Van Alkemade, 2014).

At the same time, the excessive growth of hospital expenditure urged the government to conclude ‘General agreements’ with the hospital and insurer associations to limit total spending growth of the hospital sector. Initially, for the period 2012–2013, the annual growth limit was set at 2.5% in real terms (i.e. excluding wage and price adjustments), but in subsequent years, the maximum growth rate was reduced to 1.5% (for 2014), 1.0% (for 2015–2017) and 1.4% (for 2018). To be able to enforce the agreed upon growth limits, the government created a new legal ‘macro budget control instrument’. This instrument empowers the government to compensate any total budget overrun by imposing a levy on each hospital in proportion to its revenue. To date, however, this instrument only functioned as a credible threat but has not been used in practice, since total hospital spending growth – except for a small exceedance in 2013 – remained within the stated limits.

The General agreements also affected the contractual agreements between hospitals and insurers as they started to negotiate fixed or maximum lumpsum payments based on previous years' expenditures (Ruwaard *et al*., 2014). Prices of DTCs were used as a payment mechanism to collect the lumpsum amount (implying zero DTC prices after the agreed upon lumpsum payment level being reached). These lumpsum payments did not only provide both parties with more certainty about revenues (hospitals) and expenses (insurers), but also made it possible to provide the government with sufficient certainty that total hospital expenditure would remain within the agreed upon macro budget.

In 2015, the DTC system was again substantially changed by two measures. First, the remuneration of medical specialists was integrated in the DTC prices, implying that specialists were no longer paid by the insurers but by the hospital in which they are working. Hitherto, medical specialists were paid a regulated fee per DTC (based on a regulated payment per hour and a normative amount of time per DTC). Second, the maximum duration of a DTC was lowered from 365 to 120 days.

Since 2012, payment negotiations between hospitals and insurers typically consist of two consecutive stages (Van Rooy, 2014). First, both parties negotiate an annual maximum or fixed lumpsum payment (which may be adjusted during the year due to unforeseen circumstances). Next, individual or a bundle of DTC prices are negotiated for hospital products included in the free pricing segment.

In sum, since the introduction of the health care reform, the hospital payment system has been frequently and profoundly changed which caused considerable uncertainty among both hospitals and insurers about what would be the appropriate prices for DTCs.

## 3. Data and method

The variation in hospital contract prices may be due to a combination of hospital pricing factors and bargaining efforts by insurers. However, little research has been done to study this price variation of hospital products across hospitals and insurers in the Netherlands. One problem here is that contract prices are private information and, therefore, difficult to obtain. Recently, however, this information has been made partially available by a large health insurer (*CZ*), with a market share of 21% in 2016 (NZa, 2016), and a medium-sized hospital (*IJsselmeerziekenhuizen*), a general hospital with 360 beds. This allowed us for the first time getting insight in the extent of price variation across hospitals and the extent of within-hospital price variation across insurers in the free pricing segment.

From an insurer perspective, we studied price variation between hospitals within insurer *CZ*. The contract prices published by insurer *CZ* in 2016 include all hospital products that were priced by at least one hospital below the maximum deductible level for consumers. For consumers, only prices below the maximum deductible level are relevant because people have to pay health care expenses out-of-pocket until the deductible level is reached. In 2016, there was a mandatory deductible of €385/year for all adult people (18 years and over). In addition, these people could also opt for a voluntary deductible up to €500/year in return for a premium discount. Hence, the maximum out-of-pocket expenses per individual are €885/year. Because variation in hospital prices below €885 may matter for consumers, consumer organizations put pressure on insurers and hospitals to release information about these prices. We compare contract prices of about 1400 individual hospital products negotiated by *CZ* with three university hospitals, 56 general hospitals and 178 independent treatment centers (ITCs) (CZ, 2016). In the Netherlands, ITCs may offer only routine-type elective care for which no hospital admission is required, comprising the relatively inexpensive DTCs that are included in this dataset. The dataset includes about 70,000 prices of individual products up till a price of €2847.

We analyze price variation across insurers within a hospital, using contract prices published by *IJsselmeerziekenhuizen* (MC Groep, 2016) as negotiated with all health insurers, including five health insurance companies (*CZ*, *DSW*, *Menzis*, *VGZ*, *ZK*), and a purchasing cooperative of several small health insurers (*Multizorg*). After excluding all DTC prices that were not freely negotiable (i.e. regulated by the NZa), the dataset includes about 20,000 contract prices for 3941 hospital products (DTCs) with a maximum price of €55,080.

We provide insight into the variation of hospital contract prices in the following ways. We first construct several graphs to visualize the price variation across hospitals for all hospital products and for various subsets of products (i.e. different specialties). To this end, we rank all individual hospital products by the average price per product (averaged over all hospitals), from low to high contract prices, and show for each product both the average price and all contract prices. In addition, we show for each product by which factor the lowest and highest price per product differs. Next, we measure the degree to which hospitals have the same relative ranking in prices for two consecutively ranked products (by average price), by calculating the Pearson's correlation coefficients between these two products:
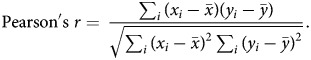


In the formula, 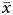 and 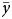 are the means of prices of two consecutive products, and *x*_*i*_ and *y*_*i*_ are the prices of these products for hospital *i*. If hospitals follow the same ranking in price for consecutive products, then *r* will be close to 1. By contrast, if hospital prices are set randomly, *r* will be around 0. Negative correlation coefficients occur when many hospitals have relatively low prices for one product but relatively high prices for the other product (in case of an exactly opposite ranking of both product prices, *r* will be close to −1). Finally, we take a closer look at the variation of contract prices for different types of hospitals and different insurers.

## 4. Results

### 4.1 Price variation across hospitals within an insurer

**Figure 1.**
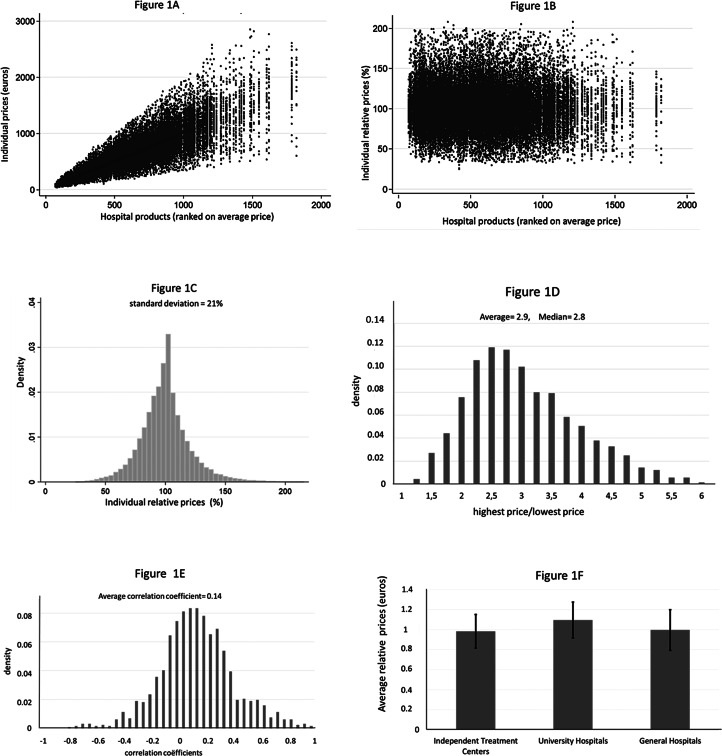
Price variation of all products contracted between insurer CZ and all hospitals. *NOTE*: 1(a) shows on the horizontal axis the individual hospital products ranked by the average price per product (averaged over all hospitals), from low to high contract prices. The individual contract prices are ranked on the vertical axis. 1(b) shows the relative price on the vertical axis where we scaled the average relative price at 1 (or 100%). 1(c) shows the distribution of all relative prices. 1(d) shows the distribution of all factors, where a factor indicates for each product the price of the highest priced hospital divided by the price of the lowest priced hospital. The first bar provides the weight of all factors that fall between 1 and 1.25, the second bar between 1.25 and 1.5, etc. 1(e) shows the distribution of the correlation coefficients for all hospital prices of two consecutive products on the horizontal axis in 1(a). 1(f) shows the average relative price for three groups of hospitals. First, we computed for each product of all three groups of hospitals the average relative prices (compared with the average price for all hospitals). Next, we computed the average over all these average relative prices. The black vertical lines within the bars with average relative prices per hospital type represent plus and minus one the standard deviation (which is the average of the standard deviations of the relative prices for all).

Figure 1 visualizes the data of all contract prices concluded by insurer *CZ* with all 260 providers (i.e. general hospitals, university hospitals and ITCs; hereafter simply referred to as hospitals). Figure 1(a) shows all prices ranked by the average price per product across all hospitals. The data show large price variations between hospitals for the same hospital product. Furthermore, the absolute price variation increases with the contract price level [Figure 1(a)]. By contrast, the relative price variation is about constant, as prices vary from about 30 to 200% of the average price [Figure 1(b)]. The constant relative price variation suggests that price competition does not differ substantially between low and high priced products. Figure 1(c) shows the distribution of all relative prices (the standard deviation being 21%). About 27% of the contract prices for a hospital product are at least 20% higher or lower than the average contract price in the market. Figure 1(d) shows that price differences can be very large. For about half of the products, contract prices between the most expensive and cheapest hospital can differ by a factor of three to six. Thus, patient hospital choice can have a huge impact on the price paid by insurer *CZ*, and potentially also for the price to be paid by the patient, insofar prices do not exceed the patient's deductible. To provide an example of such an extreme price difference, insurer *CZ* pays a contract price of €747.55 to hospital *Catharinaziekenhuis* for DTC ‘one or two outpatient clinic visits for a condition of the arteries’, which is about five times as much as *CZ* pays for the same DTC to hospital *Reinier de Graaf Gasthuis* (€156.98).

An interesting question is whether contract prices of individual products are across-the-board more expensive in some hospitals than in other hospitals. In Figure 1(e), we show correlation coefficients of consecutive products on the horizontal axis to answer this question. The correlation coefficients are symmetrically distributed around a mean of 0.14, suggesting little structure in hospital price rankings. We performed the above calculations also for two large specialisms, such as surgery and internal medicine with quite similar results (see Appendix A). In Figure 1(f), we computed the average relative prices of three different categories of hospitals: university and general hospitals and ITCs. The three university hospitals have on average about 10% higher prices for the same hospital products. Price variation is slightly lower for ITCs (SD = 17%) than for university hospitals (SD = 18%) and general hospitals (SD = 20%).

### 4.2 Price variation across insurers within a hospital

**Figure 2.**
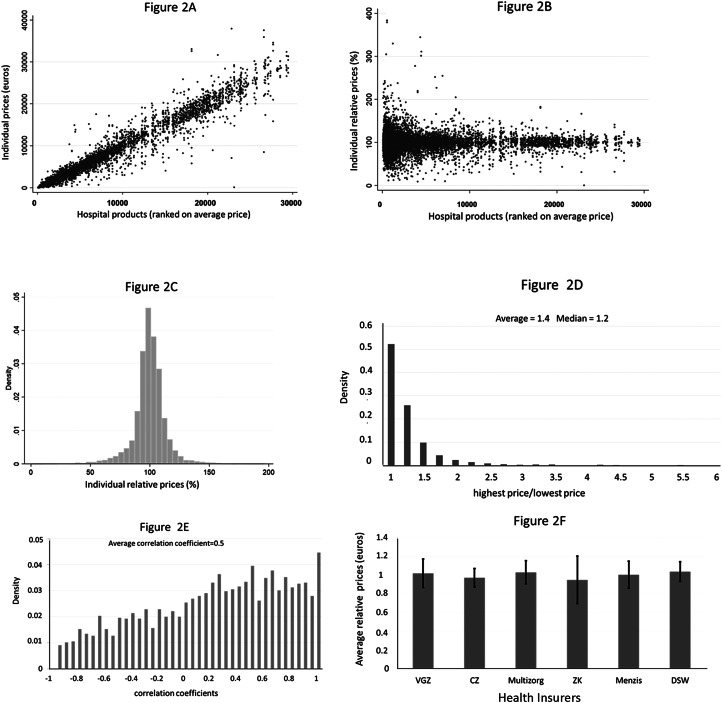
Price variation between insurers for all products offered by hospital *IJsselmeerziekenhuizen*. *Note*: 2(a) shows on the horizontal axis the individual hospital products ranked by the average price per product (averaged over all insurers), from low to high prices (until €30,000). The individual contract prices are ranked on the vertical axis. 2(b) shows the relative price on the vertical axis where we scaled the average relative price at 1 (or 100%). 2(c) shows the distribution of all relative prices. 2(d) shows the distribution of all factors, where a factor indicates for each product how much more the highest priced insurer pays as compared to the lowest priced insurer. The first bar shows all factors that fall between 1 and 1.25, the second bar between 1.25 and 1.5, etc. 2(e) shows the distribution of the correlation coefficients for all hospital prices of two consecutive products on the horizontal axis in 2(a). We only calculated correlation coefficients if a contract price existed for three or more insurers. 2(f) shows the average relative prices for six health insurers in the market. First, we computed for each product average relative prices per insurer. Next, we computed for each insurer the average of all the relative prices. The black vertical lines within the bars with relative average prices represent plus and minus one the standard deviation of all the relative prices per insurer.

Figure 2 shows, in the same way as in Figure 1, the variation in contract prices of hospital *IJsselmeerziekenhuizen* for insurers *CZ*, *DSW*, *Menzis*, *VGZ*, *ZK* and *Multizorg*. Notice, however, that the price range in the hospital dataset is much larger (ranging from about zero to €30,000) than in the insurer dataset in Figure 1 (ranging from about zero to less than €3000) because insurer *CZ* only published price data in the relevant range for consumers (i.e. if prices for at least one hospital were below the maximum deductible). Moreover, for each product, there is only a maximum of six contract prices given that there are only six different insurers in the hospital's dataset (instead of a maximum of 260 prices per product in the insurer's dataset). Figure 2(a) provides evidence of price discrimination – insurers pay a different price for the same hospital product. Figure 2(b) shows that the relative price variation decreases with the average price per product, especially for prices exceeding €3000. This suggests that insurers are willing to accept higher price variation for lower-priced DTCs, while they are more critical about the price setting of (very) expensive DTCs. Figure 2(c) indicates that the price variation in the hospital's dataset is smaller with much less outliers than in the insurer's dataset (the standard deviation being 12%). From Figure 2(d) follows that for more than 50% of the prices, the difference between the highest and the lowest insurer prices is lower than 1.25, and for 10% of the products, this difference exceeds a factor of 1.4. Price differences across insurers can be quite substantial. For example, insurer *VGZ* pays €1697 for patients receiving an ‘operation for lower back hernia’ while *CZ* pays €712. Interestingly, for only 1.7% of the hospital products, prices do not differ across insurers, which suggest that active negotiations or budget recalculations between insurers and hospitals almost always result in some price variation. Further investigation of the data, not shown here, shows that the degree of price variation is quite similar for different groups of DTCs (i.e. different medical specialisms).

Interestingly, the correlation coefficients in Figure 2(e) show a different pattern as in Figure 1 and seem to follow a more uniform distribution around a mean of 0.5. This positive correlation indicates that some insurers might obtain cheaper products than other insurers. This is confirmed in Figure 2(f). The dominant insurer in the region of hospital *IJsselmeerziekenhuizen* is insurer *Zilveren Kruis (ZK)*. This insurer negotiated on average about 5% lower prices than the average contract prices in the market, although the large standard deviation indicates that especially for this insurer the variation in relative prices is large.

## 5. Discussion

The publication of (part of) hospital contract prices by a large insurer and a medium-sized hospital in 2016 made it possible to obtain a first insight in the effect of price negotiations on prices of Dutch hospital products.

We hypothesized that effective hospital–insurer negotiations would result in limited price variation across hospitals for the same product. Contrary to our hypothesis, however, we find evidence of high and seemingly random price variation. This result is consistent for different types of hospitals and subsets of hospital products. The large price variation and low correlation of contract prices for similar products across hospitals suggest that hospitals cross-subsidize a lot of their products. The presence of substantial cross-subsidies casts doubt about the effectiveness of price competition in the Dutch hospital market, since in a competitive market the room for cross-subsidization is likely to be small because profit maximizing hospitals would have incentives to increase prices or cut back production of unprofitable products.

There are several potential explanations for these notable findings. First, hospitals are using different cost pricing methods to assign indirect costs to the various hospital products. In 2005, after the introduction of the new system product classification, hospitals were required to use a uniform cost pricing model to calculate the unit costs of DTCs (Oostenbrink and Rutten, 2006). Nevertheless, the calculation of unit costs of DTCs may differ across hospitals because hospitals may differ in how they use the uniform cost pricing model. For example, the model leaves room for discretion in how to allocate fixed costs, such as depreciation cost of buildings, inventories and technological equipment among different products.

Second, hospital prices may also differ because of different technology, wage levels, quality and efficiency of a hospital (including economies of scale and scope), and case-mix differences within DTCs.

Third, price variation can also stem from different price–cost margins per DTC as a result of differences in hospital strategy and hospital market power. Hospital pricing strategies may differ, for example, because hospitals may follow aggressive pricing strategies for specific DTCs if they want to compete with specialized hospitals or independent outpatient clinics that are located in their local market (Heijink *et al*., 2013). Differences in hospital market power may also influence the outcome of price negotiations (Oostenbrink and Rutten, 2006). Cooper *et al*. (2015) find that hospital market power is the most important observable factor in explaining hospital price variation in the US. For the Netherlands, Halbersma *et al*. (2011) found evidence of a significant positive effect of hospital market share on price–cost margins. Due to horizontal consolidation, the hospital market has become highly concentrated over the years (Schut and Varkevisser, 2017). A recent investigation by the Dutch competition authority found evidence of increasing hospital prices due to hospital mergers over the period 2007–2014 and of a positive association between hospital prices and market concentration (ACM, 2017).

A fourth explanation for the large price variation may be the considerable uncertainty about underlying costs per DTC product due to frequent changes in the product classification system and other changes in the hospital payment system (see Table 1). These frequent regulatory changes make it difficult to create a stable data infrastructure to calculate product-related cost prices. Since the introduction of negotiable prices in 2005, almost every year the payment system was substantially adjusted, frustrating adequate cost calculations.

The above explanations can explain why we find substantive price variation across hospitals but do not explain why we find price discrimination between insurers. We offer two potential explanations for this. First, since 2012, hospitals and insurers seem to have primarily focused on negotiating fixed or maximum annual lumpsum payments for all hospital products, as both parties wanted to create certainty about revenues (hospitals) and expenses (insurers) and to prevent government intervention. Hence, the contract prices may have primarily functioned as a vehicle to generate the agreed lumpsum payments. That is, given the considerable uncertainty about underlying cost prices, hospital contract prices may have been ‘mechanically’ adjusted annually to generate the lumpsum payments as negotiated by the insurer and the hospital.

A second explanation for the observed price discrimination is a combination of different purchasing strategies and differences in market power between health insurers. Moriya *et al*. (2010) find that increases in insurance market concentration in the US are significantly associated with decreases in hospital prices. A hypothetical merger between two of five equally sized insurers is estimated to decrease hospital prices by 6.7%. For the Netherlands, Halbersma *et al*. (2011) also find a significant negative impact of insurer market share on hospital price–cost margins. If hospitals also have market power, they may be able to price discriminate between insurers, particularly if insurers vary in buying power. Given that almost all hospital markets in the Netherlands are highly concentrated and regional market shares of health insurers vary widely, this is likely to be the case. Indeed, insurer market power may explain our finding that contract prices of hospital *IJsselmeerziekenhuizen* are on average somewhat lower for insurer *Zilveren Kruis* since this is by far the largest insurer in its catchment area with a regional market share of about 60% in 2016 (SFK, 2016). However, more research is needed here because the lower contract prices could also be explained by other, more random factors, such as a mechanical adjustment of contract prices.

Our study has several limitations. Due to the fact that only one insurer and one hospital published data on hospital contract prices, we observe only a limited subset of the market. This is particularly true in case of the hospital dataset, and therefore these findings should be interpreted with more caution. Since these data were only recently published, prices could also not be evaluated over time. In the future, however, more information may become available to extend our analysis. For example, two other large health insurers recently published data on hospital contract prices, albeit these price data are not easily comparable because of the use of web-tools. Another limitation of our study is that data on important factors that may explain price variation, such as differences in quality, output, treatment intensity and market shares of individual hospitals and health insurers, were not available. Given these data limitations, this study can only provide a first and largely descriptive overview of contract price variation in Dutch hospitals. Our observations may be valuable, however as a first step toward a better understanding of price setting in hospital markets where prices are freely negotiable between insurers and hospitals. The role of individual pricing factors in these markets is an interesting area for further research, as well as variation of prices over time.

## 6. Policy implications

An important policy question is whether the observed large price variation is a problem. One could argue that if hospitals and insurers effectively negotiate on maximum lumpsum payments, the underlying price variation involving cross-subsidies may not be an important problem. In the short run, competition primarily based on global budget negotiations may be effective given the prevailing uncertainty in the market. In the long run, however, as hospitals acquire better information on the underlying cost structure, systematic cross-subsidies are likely to distort incentives. Hospitals may be tempted to reduce investments in unprofitable products (the ‘bleeders’) while increasing investments in profitable ones (the ‘feeders’). As long as insurers do not have similar information on the various price–cost margins, this may result in inefficient market outcomes. The government may reduce (regulatory) uncertainty by keeping the changes in payment and product classification system as small as possible for a considerable period of time. This may enable hospitals and insurers to generate adequate information to set prices based on ‘true’ cost prices. Another potential problem of cross-subsidies is that it may complicate market regulation. If price–cost margins vary in unpredictable ways, then it becomes more difficult for a regulator to judge, for example, whether hospital prices are excessively high or low.

The large variation in hospital contract prices due to cross-subsidies may also pose a problem for consumers, insofar as these prices are below their deductible. In 2016, most people (88%) did not opt for a voluntary deductible, so for them only hospital prices below the mandatory deductible of €385 were relevant (Vektis, 2016). For a minority of 8.5% that opted for the maximum voluntary deductible of €500, hospital price variation was relevant up to €885. Until 2016, consumers were not able to base their hospital choice on the prices they had to pay because hospital price information was not publicly available. For this reason, several consumer organization urged insurers and hospitals to publish these data. A similar problem occurred in the US, where hospital prices also substantially vary and are largely opaque to the consumers (Reinhardt, 2006). Understandable hospital price information is even more relevant for US than for Dutch consumers, since US consumers typically face much higher deductibles and copayments. In order to improve consumer information on hospital prices, more than half of US states have passed legislation establishing price transparency websites or mandating health plans and hospitals to make price information available to consumers (Desai *et al*., 2016). In response to these requirements, health plans and employers have provided their enrollees or employees with online price transparency tools. As expected, people having a high deductible are more likely to use these tools. Despite the proliferation of these tools, however, most consumers do not use them and are not even aware of their existence (Gourevitch *et al*., 2017). Hence, Desai *et al*. (2016) find that offering a price transparency tool to employees was not associated with lower health spending.

**Figure 3.**
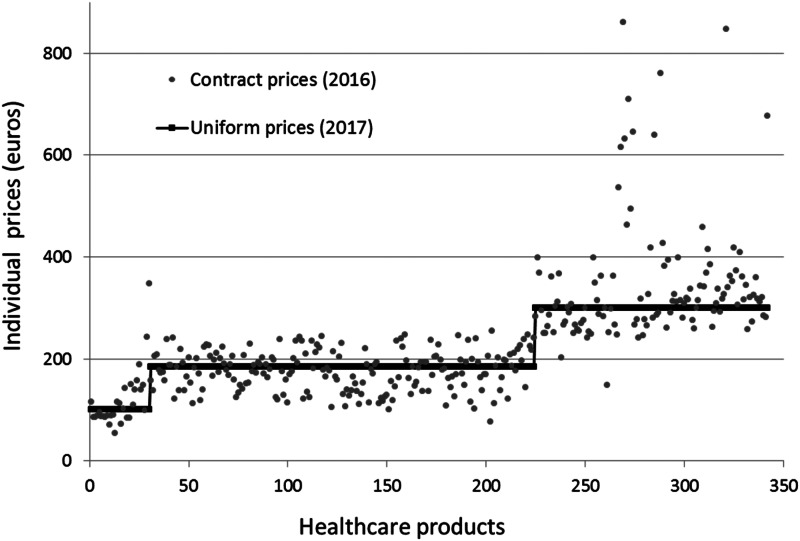
Contract prices in 2016 and 2017 negotiated between insurer *CZ* and hospital *Elkerliekziekenhuis*. *Note*: This graph shows how the contract prices for each individual product in 2016 were replaced by three levels of uniform contract prices in 2017. On the horizontal axis we ranked contract prices (2016) arbitrarily within each of the three groups of products with uniform contract prices (€100, €185 and €300).

In case of the Netherlands, it is also very unlikely that the public release of contract prices will help consumers to make price conscious hospital choices because of the multitude and complexity of the hospital products. Since DTCs represent a bundle of services, it is very hard for consumers to figure out upfront which DTC product they will receive. The idea of managed competition, however, is not about consumers shopping for lower hospital prices but about insurers purchasing high-quality care at a reasonable price on behalf of their consumers. This implies that consumers have to visit the providers in the provider network of their insurer. Prices below the deductible for similar hospital products can therefore be the same for all providers in the network. The public discontent about non-transparent hospital prices may urge hospitals and insurers to simplify the hospital pricing system for consumers for prices below the deductible. In 2017, the hospital *Elkerliekziekenhuis* introduced a system of only three uniform contract prices for 900 DTCs that were priced below the maximum deductible of €885 (*Elkerliekziekenhuis, 2017*). In Figure 3, we graphically show how these three prices relate to the contract prices for the same hospital products negotiated by insurer *CZ* in 2016. Another recent initiative to simplify consumer choice was taken by insurer Menzis who negotiated with 17 hospitals only 10 different prices for 250 DTCs below €885 (FD, 2017). These initiatives are examples from the market to make prices more uniform and comparable for consumers. However, if more insurers and hospitals come up with these kinds of initiatives, the market may still end up in a very complicated choice setting. Therefore, the government could play an important role in coordinating this process by requiring hospitals and insurers to use similar subsets of DTC products for which consumers would have to pay the same maximum price (or copayment).

If the large price variations and cross-subsidies remain, then the regulator might intervene in the market. One policy option would be to reduce price variation by using reference pricing. Reference pricing does not necessarily diminish cross-subsidizations within hospitals but prices are likely to become more transparent for insurers and consumers which may positively affect price competition (see e.g. Whaley and Brown, 2018). In this case, the regulator may set a reference price for each DTC, for example, the average price in the market. Reference pricing may also stimulate hospitals and insurers to negotiate about a hospital-specific monetary conversion factor for individual or a bundle of products, for example, all products within a medical specialism (Reinhardt, 2006). The larger the bundle the fewer conversion factors that are necessary. The reference price plus the conversion factor determine the contract price for each product. A higher conversion factor might reflect, for example, higher quality, higher treatment intensity or more inefficiency. In this case, insurers negotiate with hospitals not about each single product but about composite goods. However, more uniform pricing may also reduce the scope for optimal price negotiation and may result in less efficient outcomes (Laffont and Tirole, 1993). Hence, the most appropriate way of hospital price setting still is an open question.

## Figures and Tables

**Figure A1.**
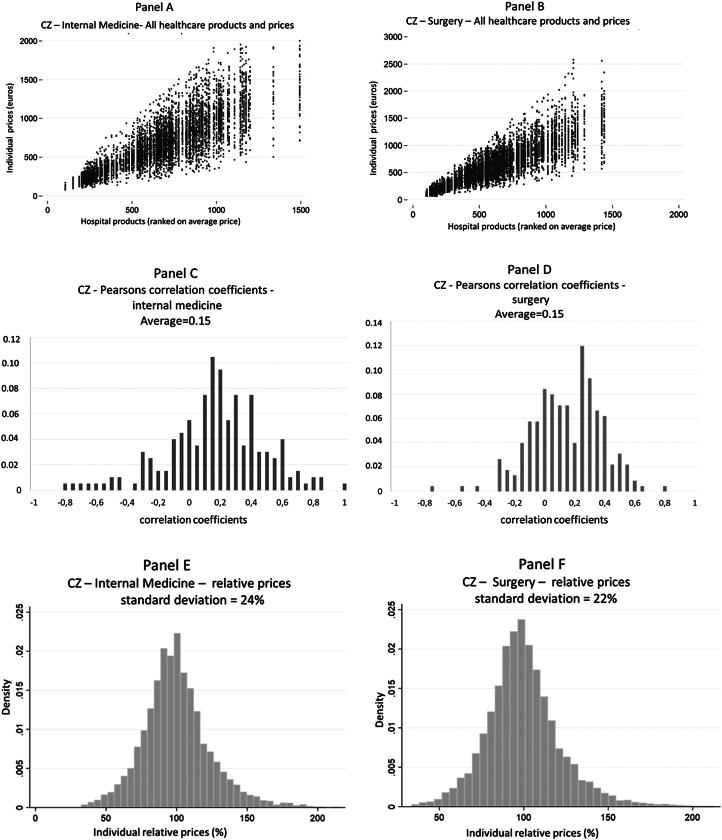
Price variation from the insurer perspective, for internal medicine and surgery. *Note*: Panel A and B show on the horizontal axis the individual hospital products ranked by the average price per product (averaged over all hospitals), from low to high contract prices. The individual contract prices for each hospital are ranked on the vertical axis. Panel C and D show the distribution of the correlation coefficients for all hospital prices of two consecutive products on the horizontal axis in Panel A and B. Panel E and F show the distribution of all individual relative prices where we scaled the average relative price at 1 (or 100%).
